# Electronic Structure and Band Gap Engineering of Two-Dimensional Octagon-Nitrogene

**DOI:** 10.1038/s41598-018-19496-7

**Published:** 2018-01-26

**Authors:** Wanxing Lin, Jiesen Li, Weiliang Wang, Shi-Dong Liang, Dao-Xin Yao

**Affiliations:** 1 State Key Laboratory of Optoelectronic Materials and Technologies, School of Physics, Sun Yat-Sen University, Guangzhou, P. R. China; 2 School of Environment and Chemical Engineering, Foshan University, Foshan, P. R. China

## Abstract

A new phase of nitrogen with octagon structure has been predicted in our previous study, which we referred to as octagon-nitrogene (ON). In this work, we make further investigations of its stability and electronic structures. The phonon dispersion has no imaginary phonon modes, which indicates that ON is dynamically stable. Using *ab initio* molecular dynamic simulations, this structure is found to be stable up to room temperature and possibly higher, and ripples that are similar to that of graphene are formed on the ON sheet. Based on the density functional theory calculation, we find that single layer ON is a two-dimension wide gap semiconductor with an indirect band gap of 4.7 eV. This gap can be decreased by stacking due to the interlayer interactions. Biaxial tensile strain and perpendicular electric field can greatly influence the band structure of ON, in which the gap decreases and eventually closes as the biaxial tensile strain or the perpendicular electric field increases. In other words, both biaxial tensile strain and a perpendicular electric field can drive the insulator-to-metal transition, and thus can be used to engineer the band gap of ON. From our results, we see that ON has potential applications in many fields, including electronics, semiconductors, optics and spintronics.

## Introduction

Since the discovery of graphene, two-dimensional (2D) materials have attracted the attention of both theorists and experimentalists^1^. In the past several years, structures of new 2D materials have been proposed by theoretical prediction and confirmed by experiments^2,3^.

The research of 2D materials of group V is one of the foci in recent years^4–7^. The black phosphorus monolayer material has been investigated by first principle calculation, and prepared by mechanical exfoliation^8,9^. Using black phosphorus as a precursor, blue phosphorene, one of the three additional newly predicted phases of 2D structures of phosphorus, has been prepared by molecular beam epitaxial growth on Au(111)^10^. A stable 2D periodic atomic sheet consisting of carbon octagons, coined as octagraphene was proposed^11^, while the on-surface synthesis and electronic properties of graphene-like nanoribbons with periodically embedded four- and eight-membered rings was reported in reference^12^.

All of the monolayer of group V materials are insulators, and some of them have non-trivial topological properties. In contrast with graphene, the band structures of pnictogen monolayer can be controlled due to their intrinsic band gap. There are two techniques to control the band gap of monolayer pnictogen, either by the application of tensile strain or a perpendicular electric field^13,14^. Moreover, the band gap of the system decreases as the number of layers increases due to inter-layer couplings.

We have predicted two different structures of monolayer that consist of nitrogen atoms: honeycomb nitrogene and octagon-nitrogene (ON)^15–17^, and have investigated the existence and gap engineering of nitrogene^16^. It is interesting to notice that one zigzag ON nano-ribbon presents two linear bands, which might indicate the existence of a Dirac point^17^. In this paper, we further investigate the stability and band structure of ON. The stability of ON is further verified by phonon dispersion and first-principle molecular dynamics. In addition, we make more indepth investigation of the electronic structures. More accurate band structures from hybrid functionals are obtained, and compared with our previous results calculated using pure functionals. Moreover, the band structure under biaxial tensile strain and in the presence of a perpendicular electric field is studied in detail, and we find that the electronic structure of ON can be controlled by adjusting both the strain and the field strength. These findings show that ON may be a promising material in some electronic devices^18–22^.

## Results

### Structure and Stability

**Figure 1:**
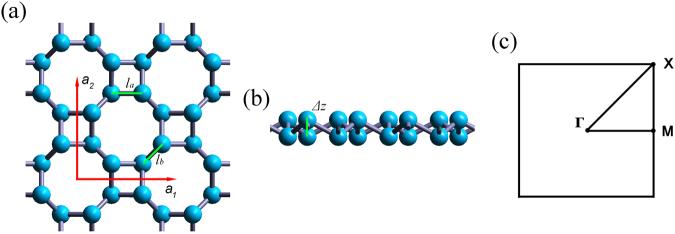
(**a**) Top view and (**b**) side view of ON. The red arrows ***a***_**1**_, ***a***_**2**_ show two basis vectors of the unit cell. (**c**) The first Brillouin zone and the high symmetry points.

Figure 1 shows the geometric structure of ON. Each cell contains eight nitrogen atoms that are not coplanar. Hence, ON has buckling structure similar to nitrogene. Here, *a* is the lattice constant, *l*_*a*_ is the length of a bond in the square, *l*_*b*_ is the length of the bond connecting two squares, as shown in Fig. 1(a), the *Δz* is the buckling distance, as shown in Fig. 1(b)^17^.

**Figure 2:**
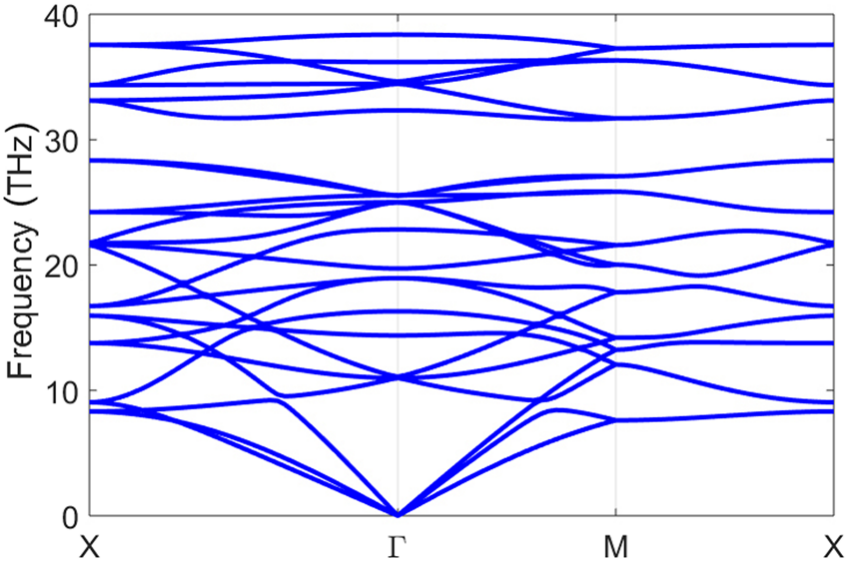
Phonon dispersion of ON monolayer.

**Figure 3:**
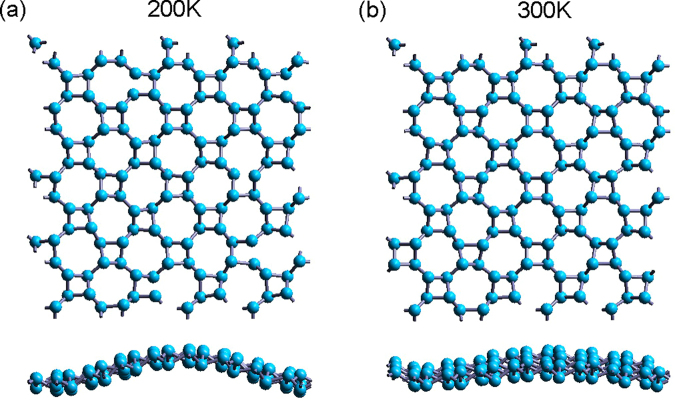
MD simulations of ON. The top and side views of the snapshots of the ON lattice structure in the MD simulations at 200 K (**a**), 300 K (**b**), respectively.

The stable geometric structure of ON has been obtained by performing structure optimization in our previous work^17^. While in this paper, in order to investigate the stability of the ON, the phonon dispersion has been calculated, and the first principle MD simulations have been performed with over 2 picoseconds at 200 K and 300 K. From the phonon dispersion in Fig. 2, ON is found to be stable because no vibration modes with imaginary frequency are presented, the lowest phonon band near Γ point is highly firm. During the evolution of MD simulations, the sheet develops ripples along one of the axes, as shown in the snapshots in Fig. 3. The 2D ON lattice is dynamically stable at 200 K and 300 K without breaking the bonds, which indicates the structure is stable at room temperature.

### Electronic Structures of Single Layer and Multilayer

**Figure 4:**
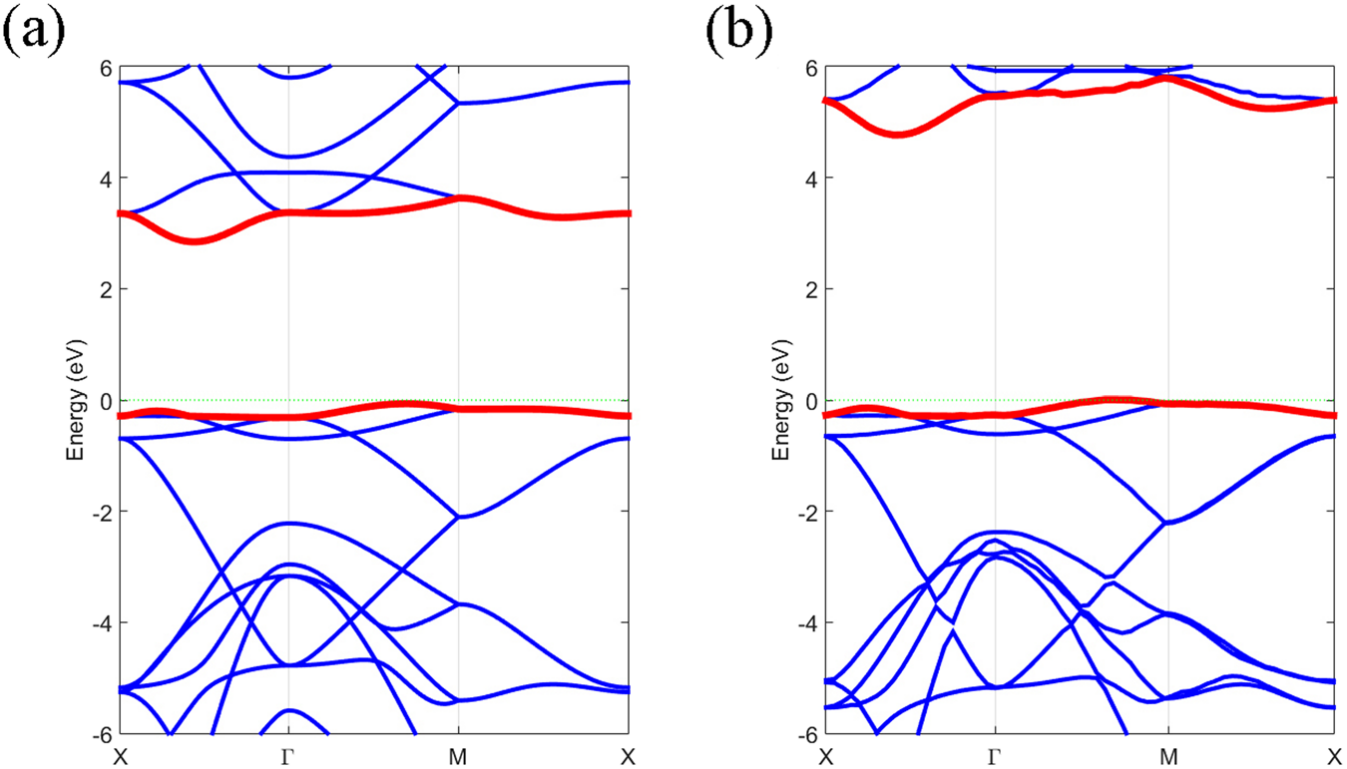
(**a**) The band structure calculated by PBE (**a**) and HSE (**b**) along high-symmetry points in the Brillouin zone. The energy is scaled with respect to the Fermi energy E_F_. The red lines denote CBM and VBM, respectively.

The bands of free ON calculated by PBE without SOC are shown in Fig. 4(a) and the indirect band gap is 2.9 eV, which is the widest gap found in the octagon monolayer of group V elements^17,23^. Since pure functional tends to underestimate the band gap, we also calculated the band structure using HSE functionals for comparison, as shown in Fig. 4(b). The band gap calculated using HSE functionals is 4.7 eV and the PBE result underestimates the gap by about 1.8 eV, even though these band structures near the Fermi level are similar. From the band of free ON, the conduction band minimum (CBM) is along the X-Γ line and the valence band maximum (VBM) is along the Γ-M line, which means that ON is an indirect gap semi-conductor.

**Figure 5:**
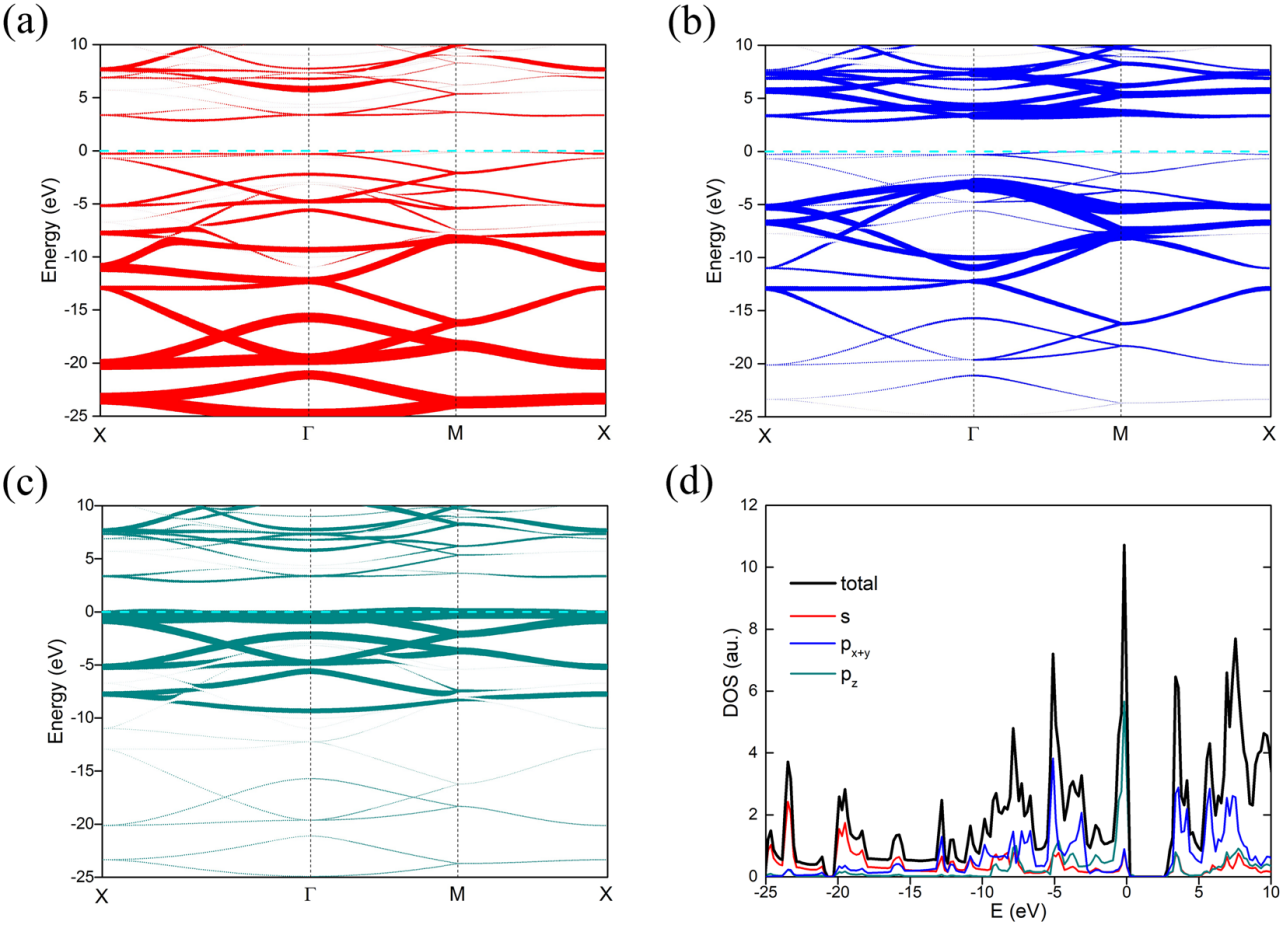
Projected electronic structure of ON. (**a**–**c**) Show the bands of s, p_x+y_ and p_z_ states, respectively, and the symbol size indicates the contribution weight. (**d**) The projected density of states of ON. Red, blue and dark cyan symbols denote the projections of the s, p_x+y_ and p_z_ states, respectively.

To further investigate the orbital feature of band structures, the projected density of states (PDOS) and projected band are calculated, as shown in Fig. 5. From Fig. 5(a–c), it is clear that the band is mainly made up of s and p orbitals, but the contributions are different. Below the Fermi level, bands in the lower energy main have more s characters, while those bands near the Fermi level have more p_z_ characters, which are responsible for the sharp peak near the Fermi energy in the density of states (DOS) as shown in Fig. 5(d). The lower band mainly consists of s orbitals, as shown in Fig. 5(a), while the contribution of p_x+y_ orbitals becomes greater as the energy increases as shown in Fig. 5(b). p_z_ orbitals play a dominant role near the Fermi level, as shown in Fig. 5(c). There is a flat band near the Fermi level, and the DOS is singular, which is mainly due to p_z_ orbitals.

**Figure 6:**
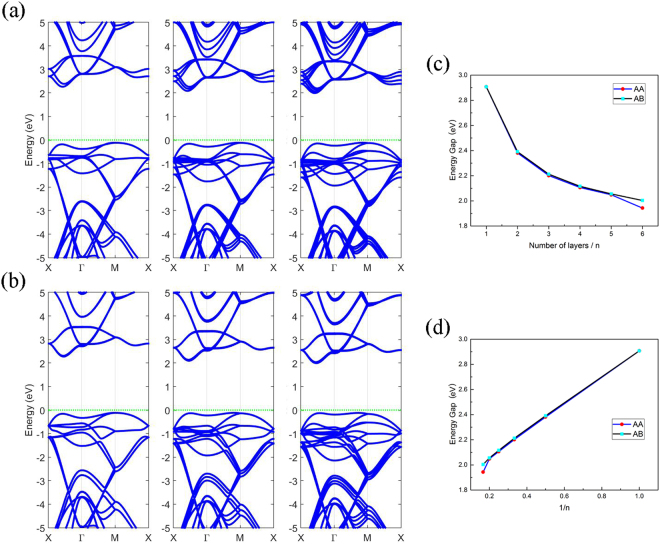
(**a**,**b**) Band structures of two, three, and four layers of ON for (**a**) AA stacking, and (**b**) AB stacking, respectively. (**c**–**d**) Show the dependence of band gap on the number of layers.

Our previous study^16^ has shown that the stacking of nitrogene can change its electronic structure. The band gap decreases as the number of layers increases and some of the degeneracy of the bands is broken, especially at some high symmetry points, as shown in Fig. 6(a,b). The gap decreases rapidly as the number of layers increases. The general trend is similar for both the AA stacking and AB stacking, see Fig. 6(c,d), and the band gap is found to be linearly dependent on the reciprocal of the number of layers.

### Effect of Biaxial Tensile Strain

**Figure 7:**
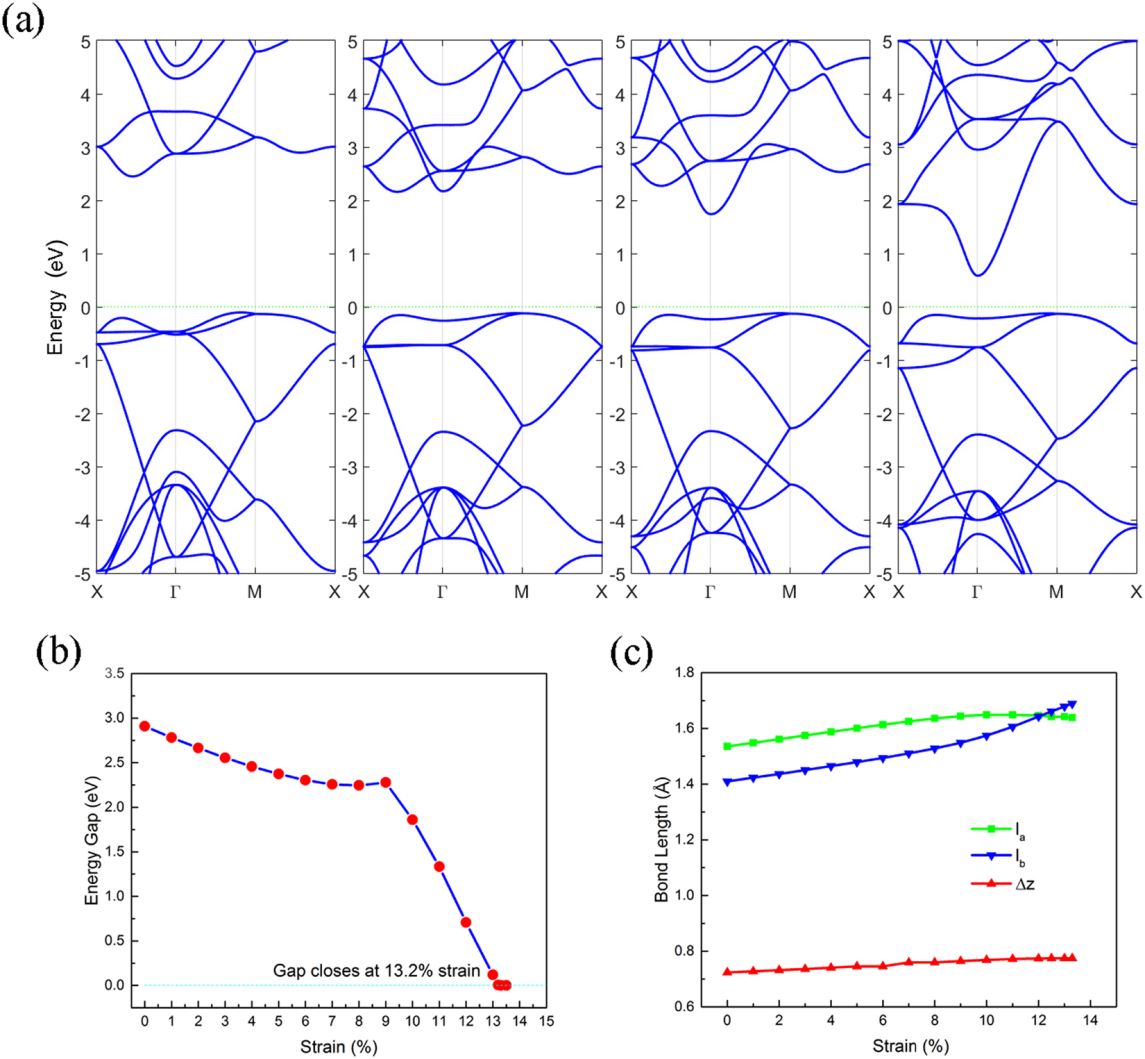
(**a**) Band structures of ON under the 3%, 9%, 10%, and 12% strains. (**b**) Dependence of energy gap on the strain. (**c**) Dependence of the covalent bond length *l*_*a*_ (green), *l*_*b*_ (blue) and buckling distance (red) on strain.

In addition to multilayer stacking, tensile strain is also a frequently used technique for manipulating the band gaps of 2D materials, because it is easier to realize experimentally. Tensile strain affects the kinetic energy of electrons, which changes the band structure directly. In the process of biaxial strain, the lattice constants have been enlarged synchronously and the atoms have been relaxed again. In Fig. 7(a), the band gap decreases with the strain, decreasing slowly in the range before 8%. Interestingly, there is a small maximum at a strain of 9%, then the gap decreases more rapidly and is almost linear as the strain exceeds 9%. The gap eventually closes at the strain of 13.2%, and the system becomes a metallic state, which is a second order phase transition. The relaxation results indicated that the ON structure was still stable at the strain of 13.2%. In the process of strain, some bands shift toward to the Fermi level. However, some bands shift up and away from it.

Figure 7(b) shows the dependence of the energy gap on strain. In the beginning, the CBM is located at a point along the X-Γ line in the Brillouin zone. The CBM then shifts to the Γ point when the strain reaches 9% or above. Figure 7(c) shows the change of structural parameters with respect to tensile stain. Without strain, *l*_*a*_ is longer than *l*_*b*_, and both *l*_*a*_ and *l*_*b*_ are monotonically increasing with respect to strain. As strain reaches 12% or more, *l*_*a*_ becomes shorter than *l*_*b*_.

### Effect of an External Electric Field

**Figure 8:**
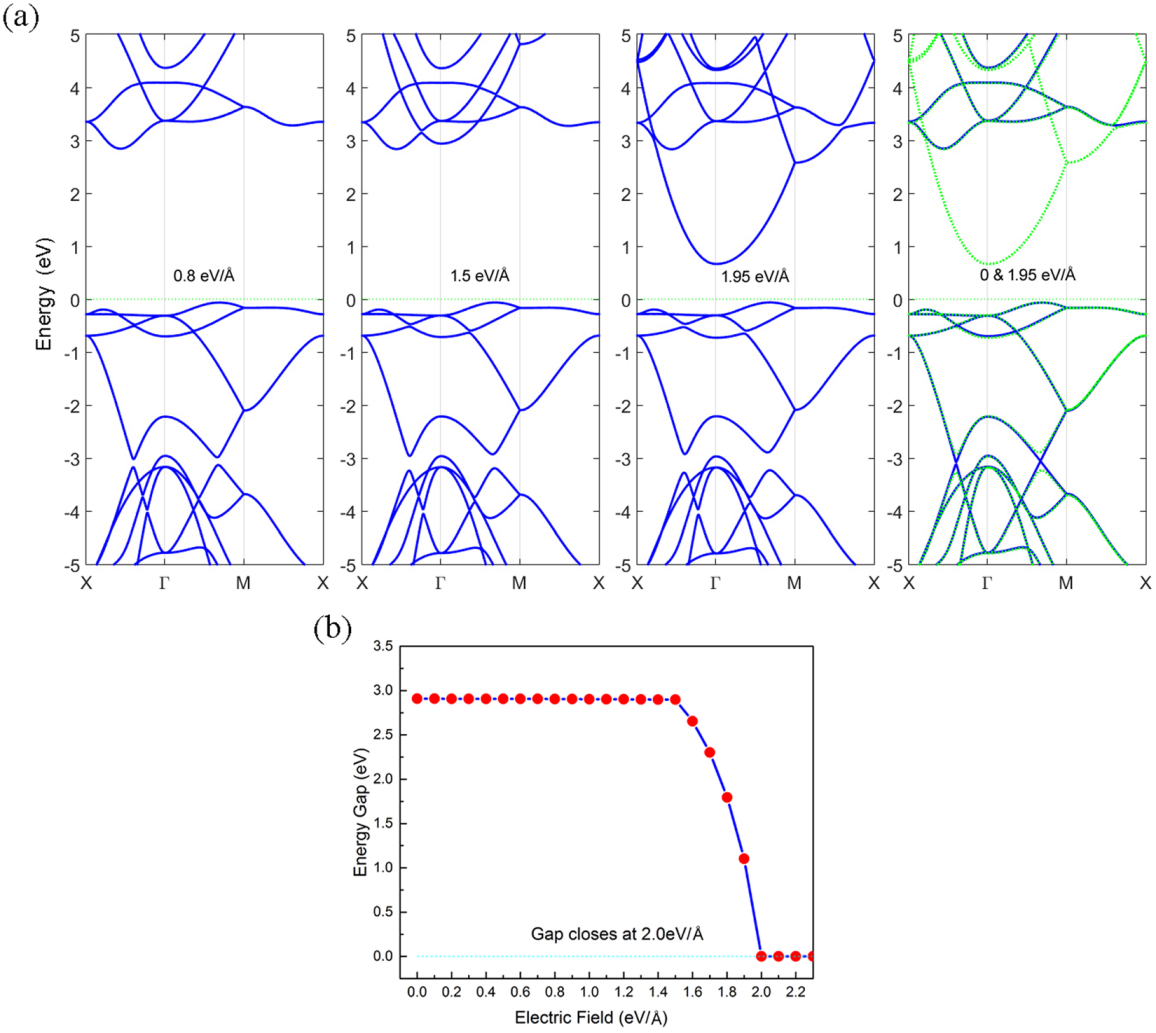
(**a**) Band structures of ON in the presence of a perpendicular electric field. (**b**) Dependence of energy gap on the field strength.

The application of an external electric field is also a useful technique to control the band structure of 2D materials. From Fig. 8(a) we can see that the band structure changes as the electric field strength increases. In the beginning, the CBM stays on the X-Γ line in the Brillouin zone but CBM shifts to Γ point when the electric field reaches 1.5 eV/Å or more. However, with the increases of the field strength, some bands shift down but some bands remain unchanged. For comparison, we plot the band structure in the absence of an external electric field and in the presence of an electric field of 1.95 V/Å, together, in the fourth panel of Fig. 8(a). The gap opens at 3 eV below Fermi level under the presence of the electric field. The band gap does not change untill the field strength reaches electric field of 1.5 eV/Å but the high energy bands move toward to the Fermi level. When the electric field reaches 1.5 eV/Å, the band gap decreases rapidly and closes at 2.0 eV/Å. The system then becomes metallic, as shown in Fig. 8(b). Some other materials^16,24,25^ such as nitrogene also show similar phenomenon, due to the electron density redistributed under the electric field^26,27^. However, the critical field for transition is much higher than for nitrogene.

## Discussion

In this study, the stability and electronic structure of ON have been studied by first-principle calculations. Phonon dispersion, as well as first-principle molecular dynamics (MD) suggest that ON is stable. The MD result shows the ON lattice is dynamically stable at 300 K without breaking the bonds, and that wraps and ripples are present at finite temperatures, which indicates the structure is stable at room temperature. Results based on the density functional theory calculation suggest that ON is a semiconductor with an indirect band gap of 2.9 eV/4.7 eV (PBE functional/HSE06 functional), which is the widest one in the octagon monolayer of group V elements. Analysis of the orbital character of the band structure is very helpful in constructing the tight-binding model, which would be beneficial for further study of its properties. In addition to monolayer ON, we also studied the electronic structure of multilayer ON. Both the AA stacking and AB stacking can decrease the band gap and have almost the same band gap for multilayer ON. Biaxial tensile strain can decrease the band gap as well, and a nearly linear dependence of gap on strain is found when the strain is between 9% and 13.2%, where the gap closes. Moreover, the perpendicular electric field can lower the energy of bands far above the Fermi level while keeping the ordinary bands intact. We therefore found that the gap remains the same at the range of from zero to 1.5 eV/Å but begins to decrease as the electric field strength reaches 1.5 eV/Å and closes at 2.0 eV/Å. Though the critical electric field is much high, this perhaps can be realized by the substrate. This study suggests that ON is a wide band gap semi-conductor and that its electronic structure can be tailored by several techniques. Our results suggest that further analysis using other methods, such as vacancy, doping and adsorption may demonstrate the existence of other interesting phenomena in the ON system, such as the existence of a Dirac cone or additional topological properties^29^. The transport properties of ON may exhibit some interesting phenomena, which could be seen by combining the density functional theory with the nonequilibrium Green’s function formalism^30^. We expect that ON can be further stabilized when assembled on a substrate, because the rotational freedom will be quenched. The real ON can possibly be synthetized by comprising non-hexagonal rings with nitrogen molecules on Au(111) surfaces. This new novel material may be expected to be of use in many fields such as electronics, semiconductors, optics and spintronics.

## Methods

The material and electronic structure of ON have been calculated using the VASP code^28^ based on PAW with PBE of exchange-correlation. The system satisfied the periodic boundary conditions with the vacuum at least 15 Å thick between the interlayer. In order to obtain the stable structure, ions have relaxed by the conjugate-gradient method until the total force on each ion is less than 0.01 eV/Å, then it is further relaxed by a quasi-Newton algorithm until the total force on each ion is less than 0.0001 eV/Å. Phonon dispersions have been calculated by RESCU with a 2 × 2 × 1 supercell^31^. In the self-consistent calculation, an 8 × 8 × 1 *k*-point mesh with Monkhorst-Pack scheme was used for sampling the Brillouin zone under the perpendicular electric field and a 20 × 20 × 1 grid with Monkhorst-Pack scheme was used for other conditions. The MD simulations have been performed by using an NVT ensemble for a 4 × 4 × 1 supercell at 200 K and 300 K. We have not considered the spin-orbit coupling during the calculation because it is negligibly small in the ON.
